# Complete Genome Sequences for 36 Canadian Salmonella enterica Serovar Typhimurium and I 1,4,[5],12:i:– Isolates from Clinical and Animal Sources

**DOI:** 10.1128/MRA.00734-20

**Published:** 2021-01-07

**Authors:** Justin Schonfeld, Clifford Clark, James Robertson, Gitanjali Arya, Shannon H. C. Eagle, Simone Gurnik, Roger Johnson, Genevieve Labbe, Kira Liu, Shaun Kernaghan, Amanda Mazzocco, Joanne MacKinnon, Kim Ziebell, John H. E. Nash

**Affiliations:** a Public Health Agency of Canada, National Microbiology Laboratory, Guelph, ON, Canada; b Public Health Agency of Canada, National Microbiology Laboratory, Winnipeg, MB, Canada; Portland State University

## Abstract

Here, we report the complete genome sequences for 36 Canadian isolates of Salmonella enterica subsp. *enterica* serovar Typhimurium and its monophasic variant I 1,4,[5]:12:i:– from both clinical and animal sources. These genome sequences will provide useful references for understanding the genetic variation within this prominent serotype.

## ANNOUNCEMENT

Salmonella enterica subsp. *enterica* Typhimurium and its monophasic variant I 1,4,[5]:12:i:– (mST) consistently rank among the most prevalent types of *Salmonella* causing human illness globally (1, 2). These serotypes are of public health concern due to the increasing prevalence of multidrug resistance phenotypes, including resistance to the last-resort antibiotic colistin (3–5). Despite being one of the most prevalent serotypes, there are few complete genomes available for this serotype from Canadian sources. Here, we present a diverse collection of Canadian *S.* Typhimurium and mST genomes from both clinical and animal sources, which will facilitate comparative genomics analyses.

Strains were selected from the National Microbiology Laboratory’s collection arising from Canadian outbreak and surveillance programs (Table 1). Samples were grown on LB plates overnight at 37°C, and genomic DNA was isolated using the Qiagen EZ1 DNA tissue kit on the Qiagen Advanced XL automated instrument with the DNA bacterial protocol, per the manufacturer’s protocol, using 190 μl of G2 buffer with 10 μl of proteinase K. Oxford Nanopore sequencing was performed at the National Microbiology Laboratory (NML) in Guelph, Ontario, Canada, using an Oxford Nanopore MinION sequencer with the default manufacturer’s protocol for rapid barcoding. Samples were prepared using SQK-RBK004 rapid barcoding kits and subsequently run on a FLO-MIN106 R9.4 flow cell. Albacore v2.3.3, available from Oxford Nanopore, was used to perform demultiplexing, base calling, and quality filtering of the raw reads. Illumina paired-end sequencing was performed with either a NextSeq 550 instrument with the NextSeq 500/550 300-cycle midoutput kit or a MiSeq instrument with the MiSeq v3 600-cycle reagent kit. Regardless of Illumina sequencing platform, all libraries were prepared using the Nextera XT DNA library preparation kit.

**TABLE 1:** Strains sequenced in this study

Isolate ID	BioSample no.	BioProject no.	GenBank accession no.	Collection date (yr-mo-day)	Province of origin^*a*^	Host species	Sample type^*b*^	Genome size (bp)	GC content (%)	Nanopore *N*_50_ (bp)	No. of Nanopore reads	No. of Illumina reads	
PNCS014851	SAMN11333145	PRJNA316728	CP038847, CP038848	2008-12-26	NS	Homo sapiens	Stool	4,948,841	52.22	6,407	51,021	901,777	
PNCS014866	SAMN11333163	PRJNA316728	CP038849	2012-08-01	NS	*Homo sapiens*	Stool	4,930,644	52.2	7,070	57,974	2,640,950	
PNCS014846	SAMN11333140	PRJNA316728	CP039558, CP039559, CP039560	2008-06-30	MB	*Homo sapiens*	Stool	5,123,430	52.91	5,069	27,850	940,766	
PNCS014847	SAMN11333141	PRJNA316728	CP039561, CP039562, CP039563	2008-07-16	NL	*Homo sapiens*	Stool	5,159,502	52.19	5,069	24,143	1,100,141	
PNCS014848	SAMN11333142	PRJNA316728	CP039564	2008-07-11	NS	*Homo sapiens*	Stool	4,996,602	52.2	6,913	13,075	1,078,339	
PNCS014849	SAMN11333143	PRJNA316728	CP039565, CP039566	2008-10-21	MB	*Homo sapiens*	Stool	4,909,026	52.22	5,034	24,444	918,745	
PNCS014850	SAMN11333144	PRJNA316728	CP039567, CP039568	2008-10-29	QC	*Homo sapiens*	Stool	4,909,329	52.22	5,285	23,673	927,055	
PNCS014852	SAMN11333146	PRJNA316728	CP039569, CP039570, CP039571	2009-01-22	QC	*Homo sapiens*	Stool	4,938,638	52.1	4,263	73,645	998,205	
PNCS014855	SAMN11333149	PRJNA316728	CP039572, CP039573, CP039574, CP039575	2010-03-11	QC	*Homo sapiens*	Stool	4,992,679	52.1	3,763	35,377	1,544,290	
PNCS014856	SAMN11333151	PRJNA316728	CP039576, CP039577, CP039578	2010-06-17	BC	*Homo sapiens*	Stool	5,083,787	52.1	4,875	46,522	992,785	
PNCS014857	SAMN11333152	PRJNA316728	CP039579, CP039580, CP039581	2010-06-09	QC	*Homo sapiens*	Stool	5,031,864	52.13	4,411	23,877	1,055,528	
PNCS014858	SAMN11333154	PRJNA316728	CP039582, CP039583, CP039584	2010-12-08	QC	*Homo sapiens*	NA	5,030,765	52.13	4,838	23,858	1,077,691	
PNCS014859	SAMN11333155	PRJNA316728	CP039585, CP039586, CP039587	2010-12-31	ON	*Homo sapiens*	Stool	4,968,015	52.1	5,889	20,854	977,794	
PNCS014861	SAMN11333157	PRJNA316728	CP039588, CP039589, CP039590	2010-11-17	ON	*Homo sapiens*	Stool	4,974,125	52.2	5,427	13,697	1,825,406	
PNCS014862	SAMN11333158	PRJNA316728	CP039591, CP039592	2011-01-19	ON	*Homo sapiens*	Stool	5,036,798	52.12	5,180	30,692	1,056,405	
PNCS014863	SAMN11333160	PRJNA316728	CP039593, CP039594	2011-01-27	BC	*Homo sapiens*	Stool	4,987,243	52.19	3,626	32,872	957,395	
PNCS014865	SAMN11333162	PRJNA316728	CP039595, CP039596, CP039597, CP039598	2012-03-08	NL	*Homo sapiens*	Stool	4,955,652	52.22	4,317	28,769	2,670,900	
PNCS014867	SAMN11333164	PRJNA316728	CP039599, CP039600, CP039601, CP039602	2012-09-07	ON	*Homo sapiens*	Stool	4,962,621	52.12	4,809	20,379	2,821,063	
PNCS014868	SAMN11333165	PRJNA316728	CP039603, CP039604, CP039605, CP039606	2012-09-17	ON	*Homo sapiens*	Stool	4,954,168	52.19	4,692	28,248	2,914,604	
PNCS014873	SAMN11333166	PRJNA316728	CP039607, CP039608, CP039609	2014-09-16	QC	*Homo sapiens*	Stool	5,005,840	52.2	5,471	46,610	3,856,228	
PNCS014875	SAMN11333167	PRJNA316728	CP039610	2014-12-06	BC	*Homo sapiens*	Stool	4,995,830	52.2	6,341	37,757	1,009,327	
PNCS014853	SAMN11333147	PRJNA316728	CP039713, CP039714, CP039715	2009-07-20	MB	*Homo sapiens*	Stool	5,006,968	52.19	6,478	16,150	958,691	
PNCS000211	SAMN11333150	PRJNA316728	CP039716, CP039717, CP039718	2010-04-30	MB	*Homo sapiens*	Stool	5,131,386	52.92	6,795	13,720	713,985	
PNCS014860	SAMN11333156	PRJNA316728	CP039719, CP039720	2010-12-16	ON	*Homo sapiens*	Stool	4,980,441	52.1	5,889	45,631	4,117,979	
PNCS014864	SAMN11333161	PRJNA316728	CP039854, CP039855	2011-01-29	BC	*Homo sapiens*	Stool	4,910,901	52.22	4,476	110,836	1,044,461	
PNCS014876	SAMN11333168	PRJNA316728	CP039856, CP039857, CP039858, CP039859	2015-02-03	SK	*Homo sapiens*	Stool	5,237,229	51.9	6,450	36,729	1,270,479	
PNCS014880	SAMN11333170	PRJNA316728	CP039860, CP039861, CP039862, CP039863, CP039864	2016-08-26	ON	*Homo sapiens*	Stool	5,346,495	51.85	5,262	38,873	992,129	
PNCS009887	SAMN06030095	PRJNA353625	CP040318, CP040319, CP040320	2011-01-13	QC	*Homo sapiens*	Stool	4,959,032	52.17	5,347	18,866	818,172	
PNCS014879	SAMN11333169	PRJNA316728	CP040321, CP040322, CP040323	2016-08-09	BC	*Homo sapiens*	Stool	5,022,883	52.12	6,948	50,770	872,249	
PNCS009880	SAMN06030081	PRJNA353625	CP040648, CP040649, CP040650	2010-11-12	QC	*Homo sapiens*	Stool	4,916,124	51.85	5,814	38,873	992,129	
SA20070548	SAMN10833327	PRJNA316728	CP040651, CP040652, CP040653	2006-12-11	QC	Sus scrofa	NA	5,013,617	52.06	8,590	247,438	618,970	
SA20082869	SAMN10833328	PRJNA316728	CP040668, CP040669, CP040670	2008-06-17	QC	*Sus scrofa*	NA	5,014,902	52.13	8,491	173,422	605,737	
SA20143792	SAMN10833329	PRJNA316728	CP041026, CP041027, CP041028	2013-11-14	AB	Bos taurus	NA	5,163,447	52.13	8,692	198,439	645,713	
PNCS007087	SAMN11333153	PRJNA316728	CP044957, CP044958, CP044959, CP044960	2010-08-02	SK	*Homo sapiens*	Stool	5,219,843	51.88	4,636	18,866	818,172	
PNCS014881	SAMN11333171	PRJNA316728	CP044961, CP044962, CP044963, CP044964, CP044965, CP044966	2016-10-01	BC	*Homo sapiens*	Stool	5,141,910	52.18	5,262	136,603	2,000,955	
PNCS007098	SAMN11333159	PRJNA316728	CP044968, CP044969	2011-01-17	SK	*Homo sapiens*	Stool	5,111,827	52.01	5,844	17,867	836,201	

a All strains were isolated in Canada. NS, Nova Scotia; MB, Manitoba; NL, Newfoundland and Labrador; QC, Quebec; BC, British Columbia; ON, Ontario; SK, Saskatchewan; AB, Alberta.

b NA, not available.

Nanopore data were assembled using Canu v1.8 (6) (useGrid=False corOutCoverage=999), and hybrid *de novo* assemblies were produced using the Unicycler pipeline v0.4.5 (7) with the default options. Canu assemblies were trimmed using berokka v0.2.3 (https://github.com/tseemann/berokka) to remove any overhangs and then were used as input back to Unicycler with the flag “--existing_long_read_assembly.” Iterative polishing of each assembly was performed sequentially using Racon v1.3.2 (8) and Pilon v1.2.2 (9) until each tool made no further changes or converged. The circularity of each contig was confirmed using the Unicycler log files. The start site for each chromosome was set with Circlator v1.5.5 (10) fixstart using the *thrA* sequence from *S.* Typhimurium strain LT2 (GenBank accession no. NC_003197). The predicted serotype was determined using the *Salmonella In Silico* Typing Resource (SISTR) (11) to confirm that the *in silico* predictions matched the phenotypic serotype. Plasmid typing was performed using MOB-suite v1.4.9 (12). Genome sequences were uploaded to NCBI and annotated using the PGAP (13).

These newly assembled closed reference genomes and plasmids will be useful for comparative genomics applications, as well as for genomic epidemiological studies of *Salmonella*.

### Data availability.

The genome sequences for the 36 isolates have been deposited in NCBI/DDBJ/ENA under BioProject no. PRJNA353625 and PRJNA316728. The GenBank accession numbers are listed in Table 1.
